# Benzodiazepines: Drugs with Chemical Skeletons Suitable for the Preparation of Metallacycles with Potential Pharmacological Activity

**DOI:** 10.3390/molecules26092796

**Published:** 2021-05-10

**Authors:** Artur V. da Silva, Simoni M. P. Meneghetti, Mario R. Meneghetti

**Affiliations:** Grupo de Catálise e Reatividade Química, Instituto de Química e Biotecnologia, Universidade Federal de Alagoas, Av. Lourival de Melo Mota, s/n, Maceió/AL 57072-900, Brazil; artur.silva@arapiraca.ufal.br (A.V.d.S.); simoni.plentz@iqb.ufal.br (S.M.P.M.)

**Keywords:** organometallic complexes, biological activity, benzodiazepines, metallodrugs

## Abstract

The synthesis of organometallic compounds with potential pharmacological activity has attracted the attention of many research groups, aiming to take advantage of aspects that the presence of the metal-carbon bond can bring to the design of new pharmaceutical drugs. In this context, we have gathered studies reported in the literature in which psychoactive benzodiazepine drugs were used as ligands in the preparation of organometallic and metal complexes and provide details on some of their biological effects. We also highlight that most commonly known benzodiazepine-based drugs display molecular features that allow the preparation of metallacycles via C-H activation. These organometallic compounds merit further attention regarding their potential biological effects, not only in terms of psychoactive drugs but also in the search for drug replacements, for example, for cancer treatments.

## 1. Introduction

**Figure 1:**
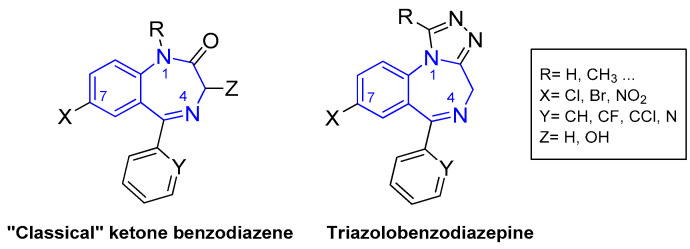
General molecular structures of the most commercialized 1,4-benzodiazepines: ketone and triazole derivatives. The 1,4-benzodizapine core is highlighted in blue.

**Figure 2:**
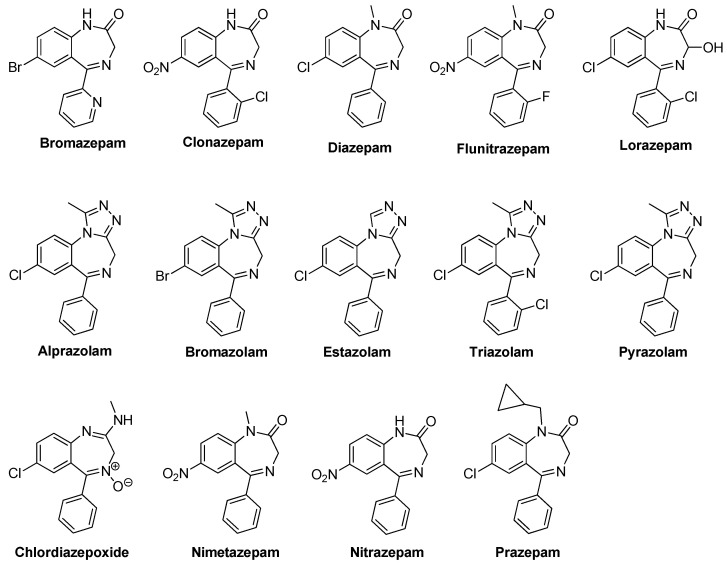
Some examples of BZDs that can be found on the market.

Benzodiazepine (BZD) drugs are a family of compounds widely used in the treatment of neurological pathologies that offer similar pharmacological properties, with anxiolytic, sedative, hypnotic, skeletal muscle relaxant, amnesic, and anticonvulsant effects [1,2], with a very well-established mechanism of action and metabolization in the organism [3]. These drugs have a chemical structure based on a core formed by the fusion of benzene and diazepine rings [4,5], and they act as positive allosteric modulators of the GABA_A_ receptor complex present in neural tissue [6,7]. They are commonly used in the treatment of sleep and anxiety disorders, with safe use in the short term [8]. Most of the pharmacologically active BZDs are 5-aryl or pyridinyl substituted on the benzo [1,4] diazepine core (see Figure 1) and the most common classes are ketone and triazole derivatives. Ketone derivatives contain a ketone oxygen bond at C(2) of the 1,4-benzodiazepine ring and are distinguished by their suffix “-azepam” Triazolobenzodiazepines contain a triazole ring condensed at the 1,4-benzodiazepine ring and are distinguished by the suffix “-zolam.” Figure 2 shows some examples of BZDs that can be found on the market [9], where some are sold as designer drugs [10].

**Figure 3:**
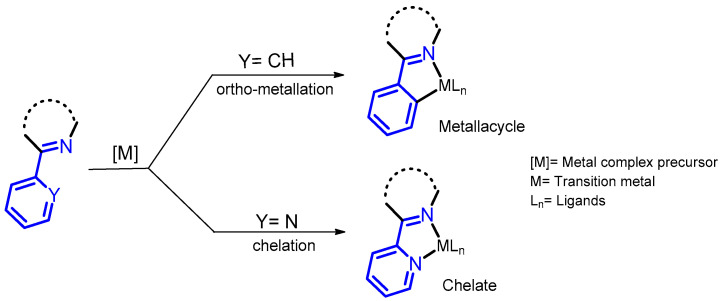
The fragments present in BZD drugs, derived from phenylmethanimine or pyridinylmethanimine, which are responsible for the reactivity of these compounds, leading to metal complexes via *ortho*-metallation or chelation.

A close look at the molecular structure of the BZDs in Figure 2 reveals why these compounds attract the attention of organometallic chemists, since they can be used as ligands in the formation of metallo-derivatives. Part of their molecular skeleton contains functional groups derived from pyridinylmethanimine or phenylmethanimine, allowing the formation of chelated metal complexes via ligand association, or *ortho*-metallation (see Figure 3). However, despite these interesting features, few studies on the transformation of BZD drugs into their metallo-derivatives have been reported.

**Figure 4:**
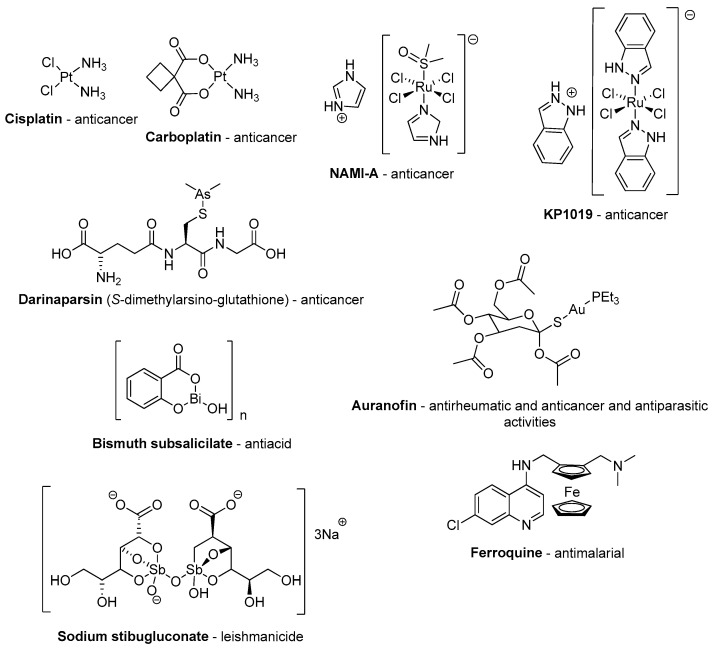
Some examples of applied metallodrugs or those in the advanced trial stage.

Prior to discussing metal derivatives of benzodiazepine drugs in detail, it should be noted that, for centuries, metal compounds have been used to treat diseases and heal wounds [11]. In the early 20th century, compounds containing arsenic, antimony, bismuth and gold were commonly prescribed to treat diseases like syphilis, leishmania, diarrhea, arthritis and others [12,13]. However, with the rapid progress in the area of organic chemistry in the last century, combined with the large-scale production of very effective new fully-organic drugs, there has been a decrease in some aspects associated with studies aimed at the discovery of new metal-based drugs. In fact, inorganic and organometallic chemists have mostly concentrated on the application of these compounds in areas like catalysis and structural chemistry. Even so, some metallodrugs have been used for specific applications (see Figure 4) and the development of these drugs has been expanding within medicinal chemistry [14]. More and more coordination and organometallic compounds are being studied, whether they are linked to organic drugs or to organic fragments that, individually, do not have pharmacological activity [15,16].

The most prominent example is the anti-cancer platinum complex cisplatin and its analogues, which, since their clinical use from the 1970s, account for about 50% of current chemotherapeutic protocols for cancer treatment [17]. Radiopharmaceuticals also play an important role in medical diagnostics and therapy [18,19]. In fact, these medical applications have trigged research to further explore inorganic and organometallic chemistry. In this regard, it should be noted that several important enzymes in our organism require metals in their structure (metalloenzymes) for their functional catalytic activity in the organism. Cytochrome P450 and plastocyanin, for instance, contain Fe and Cu, respectively, in their structure [20].

Coordination and organometallic chemistry could lead to the preparation of potential metallodrugs with wide molecular structures and chemical reactivity, i.e., with different geometries, coordination numbers, oxidation states, redox potential and ligands, which can be exploited to develop new drugs that cannot be produced using only organic compounds [21,22]. One approach to the design and development of new efficient and potent metallodrugs is based on the association of well-known organic drugs modified with the addition of the metal atoms. Here, the challenge is to maintain the pharmacological activity of the organic drug after modification, with its coordination to the metal center, since its physiological profile can be completely changed, affecting its properties and toxicity [23]. For example, the modification of an organic drug with the coordination of a metal, can significantly change the log D values of the former compound, affecting the access of the drug in tissues or organs like the brain.

In this context, this short review gathers some of the main research studies that illustrate the potential for the preparation of metallo-derivatives of BZDs, such as metallacycles and other metal complexes. In addition, their potential application as metallodrugs, taking advantage of the previously confirmed pharmacological activity provided by the ligand moiety, is discussed. Details of studies reported in the literature involving the synthesis of BZD organometallic and coordination compounds and their pharmacological applications are provided. The aim is to encourage further research involving these compounds in the field of inorganic medicinal chemistry.

## 2. Coordination and Organometallic Chemistry of BZDs

In this section, we present a historical sequence of the development of the coordination and organometallic chemistry of BZDs, with the most relevant publications summarized in Table 1.

The first studies related to the preparation of coordination compounds (adducts) containing the benzodiazepine core as ligands were published in the early 1970s, indicating the classical aspects of the coordination chemistry of the benzodiazepine core considering several metal ions [24,25]. Soon after, a series of metal-BZD adducts was prepared (see Table 1). The first examples of BZD drugs coordinated to metal ions—Co(II), Ni(II), and Cu(II)—were published by Preti and Tosi [26,27] (see Table 1, Entry 1). At that time, they suggested that the Co(II) and Ni(II) complexes could have a pseudotetrahedral symmetry, whereas the Cu(II) complexes were octahedral. In the late 1970s, the same researchers appeared to have obtained square planar BZD coordination compounds with Pd(II) and Pt(II) ions [28] (see Table 1, Entry 2). However, in 1980, more accurate analysis, using X-ray diffraction, NMR, and EPR studies, demonstrated that diazepam is, in fact, coordinated to the Cu(II) ions via its nitrogen atom N(4), leading to a *trans* square-planar complex [29] (see Table 1, Entry 3). In the early 1980s, Minghetti and coworkers prepared square planar adducts of BZDs with Au(III) [30,31] (see Table 1, Entry 4). Benedetti and coworkers obtained the first well characterized 5-member ring chelated Pt(II)-BZD complex, using bromazepam as the ligand. In this case, platinum(II) is in a square-planar environment, as expected, with bromazepam coordinated to the metal via both the imino and the pyridyl nitrogen atoms [32] (see Table 1, Entry 5). The analogous Pd(II) derivative was prepared by Antolini and coworkers [33] from K_2_PdCl_4_ as palladium (see Table 1, Entry 9).

The first organometallic BZD derivatives were reported in 1988, when Cinellu and coworkers published an important paper indicating the preparation of the adducts *trans*-L_2_PdCl_2_ (L = diazepam or prazepam) through the reaction of PdCl_2_ or (PhCN)_2_PdC1_2_ with diazepam and prazepam, in which the BZDs act as monodentate ligands through the N(4) atom. However, they obtained dimeric forms of palladacycles using the same BZDs in the presence of Na_2_PdCl_4_ (see Table 1). These palladacycles were obtained through chelation-assisted C–H activation, i.e., deprotonation of the 5-phenyl substituent (*ortho*-metallation) [34]. In addition, the dimers, with halide-bridges, were easily split in the presence of PPh_3_ or Tl(acac) [35] (see Table 1, Entry 6). At that time, Cinellu and coworkers stated that the first assignments of 1:1 BZD:Pd(II) as chelated adducts [28], in 1976, were most probably palladacycles of BZDs. It should be noted that an important publication reported the preparation of air-stable organometallic derivatives of BZDs, allowing their use in a wide range of applications [36].

**Figure 5:**
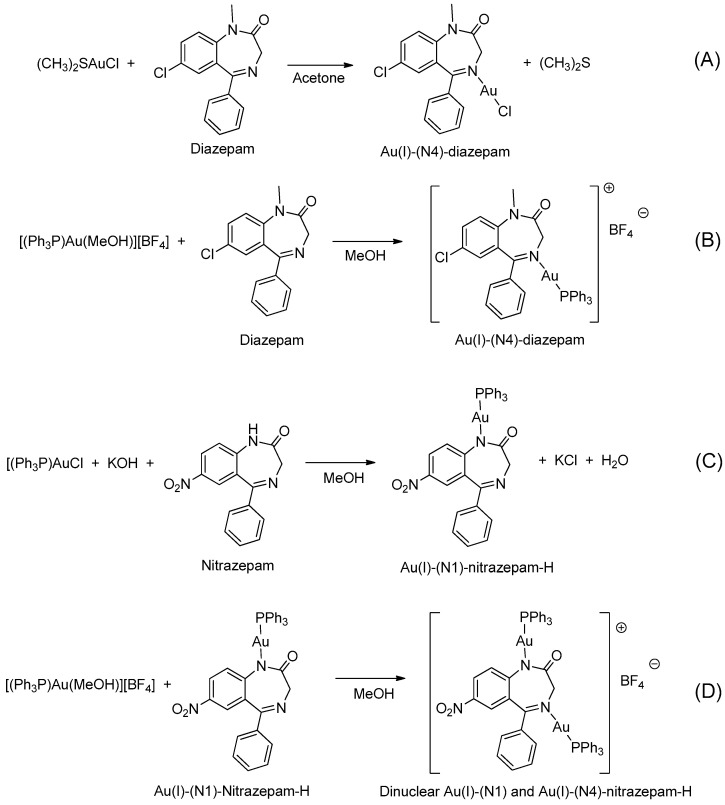
A series of BZD-Au(I) derivatives prepared by Cinellu and coworkers (**A**–**D**).

In 1990, Cinellu and coworkers prepared a series of neutral and cationic BZD-Au(I) derivatives [37], as seen in Figure 5 and Table 1, Entry 7. In this case, the BZDs were coordinated to the metal center via the imino nitrogen atom. However, in the presence of alkali, (PPh_3_)AuCl reacts with 1-unsubstituted 1,4-benzodiazepin-2-ones, i.e., nitrazepam and lorazepam, to give neutral Au(I) species (L-H)Au(PPh_3_), as shown in Figure 5C. For these neutral complexes, the coordination to the metal center occurs via the deprotonated amino nitrogen atom. The same authors also prepared a cationic species [(Ph_3_P)Au{µ-(nitrazepam-H)}Au(PPh_3_)][BF_4_] where the deprotonated nitrazepam bridges two (Ph_3_P)Au groups through the N(1) and N(4) atoms (Figure 5D). The above examples highlight the great potential of the coordination and organometallic chemistry of BZDs.

In 1991, Aversa and coworkers [38] also prepared BZD organometallic compounds derived from bromazepam. They obtained adducts of bromazepam-Pt(II), using the organometallic precursors *cis*-PtMe_2_(Me_2_SO)_2_ and [PtPh_2_(Et_2_S)]_2_ (see Table 1, Entry 8).

**Figure 6:**
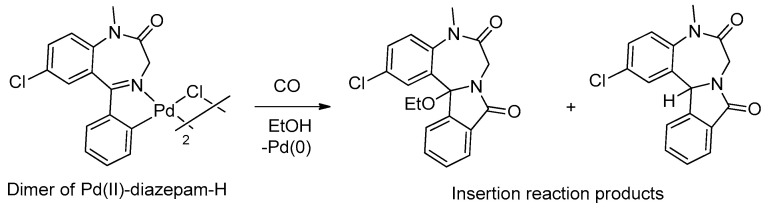
Example of an insertion reaction of CO in the Pd-C bond of palladacycles derived from BZDs.

The first results showing some aspects of the chemical reactivity of palladacycles derived from BZDs were published by Cinellu and coworkers in 1991, increasing the possibility of preparing new BZD derivatives [39]. They demonstrated that dimeric palladacycle derivatives of BZDs are able to react with carbon monoxide, leading to tetracyclic derivatives having an isoindolo ring condensed on the benzodiazepinic core. An example of this reaction is shown in Figure 6.

Similar chemistry to that observed in relation to the synthesis and reactivity of palladacycles derived from BZDs was verified for their platinacycle counterparts. In 1994, Stoccoro and coworkers [40] prepared platinacycles derived from diazepam (dimeric and monomeric forms). Interestingly, they prepared most of the monomeric forms from the monomeric complex containing two diazepam ligands, one of them cyclopalladated and the other just coordinated (see Table 1, entry 10).

**Figure 7:**
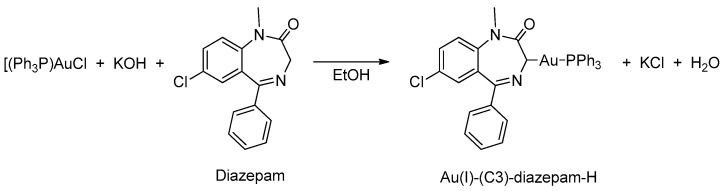
First example of organogold(I) derived from BZDs.

In 1998, Minghetti and coworkers [41] prepared the first example of organogold(I) derived from BZDs (see Table 1, Entry 11) using an approach that differed from the previously reported reactions [37]. To attain the organogold(I) derivatives, in this reaction an ethanolic solution of the base was added at room temperature, drop by drop, to a suspension of Ph_3_PAuCl and the BZD in the same solvent. In all cases, the BZDs employed were alkyl substituted in N(1), see Figure 7.

**Figure 8:**
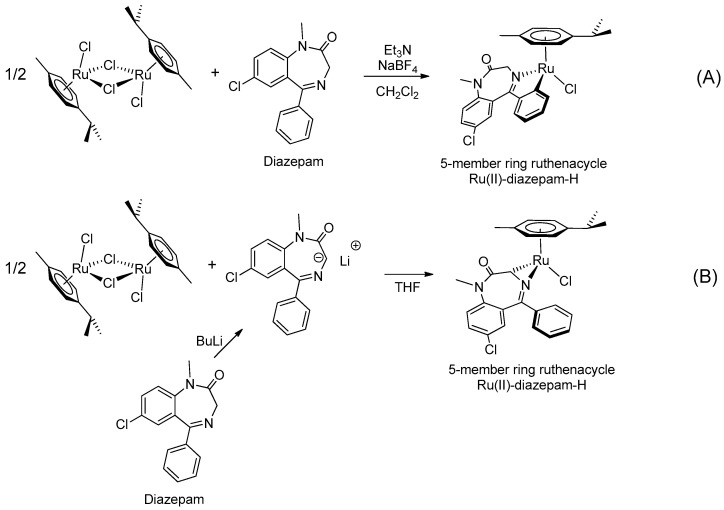
Organometallic compounds of ruthenium derived from BZDs (**A**,**B**).

In the early 2000s, Visnjevac and coworkers [42,43] demonstrated that adducts of Cu(II), Zn(II) and Pd(II) and chiral 3-substituted 5-(2′-pyridyl)-1,4-benzodiazepin-2-one derivatives can be easily attained (see Table 1, Entries 12 and 13). They report that the C(3) of the benzodiazepinic ring can undergo substitution and even ring contraction reactions [42]. Around the same time, Pérez and coworkers [44] prepared the first organometallic compounds of ruthenium derived from BZDs (see Table 1, Entry 14). They prepared 5- and 3-membered rings of ruthenacycles derived from diazepam, see Figure 8A,B.

**Figure 9:**
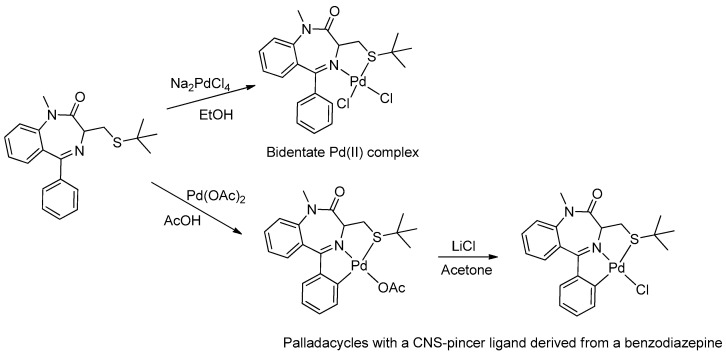
First example of a metallacycle in which the benzodiazepine derivative acts as a pincer ligand.

**Figure 10:**
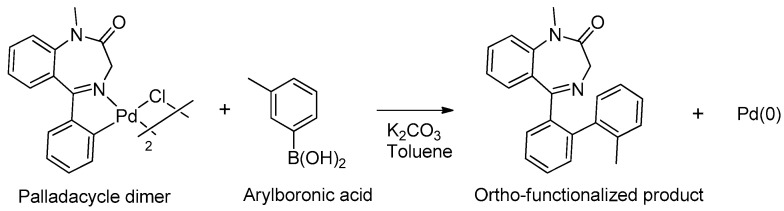
*Ortho*-functionalization of cyclopalladated 5-phenyl-1,4-benzodiazepine.

In 2005, Spencer and coworkers demonstrated that the 1,4-benzodiazepine derivatives could be used as versatile ligands for the preparation of metal complexes [45] (see Table 1, Entry 15). Through the addition of suitable substituents on the benzodiazepine core, they synthesized palladacycles via C-H activation, in which the benzodiazepine acts as a CNS-pincer ligand when using the right palladium precursor (see Figure 9). In the same publication, the authors showed that *ortho*-substituted 5-phenyl-1,4-benzodiazepin-2-ones could be produced via stoichiometric reactions of the corresponding cyclopalladated benzodiazepine in the presence of arylboronic acids (see Figure 10). Moreover, they confirmed that palladacycles of 1,4-benzodiazepine are able to catalyze Suzuki and Heck C-C coupling reactions [46].

It is important to note that most of these BZD coordination and organometallic compounds are stable in air, allowing studies on their potential application as metallodrugs. However, few examples of metallo-BZDs have been produced and not all of them have been evaluated with regard to their pharmacological potential.

## 3. Biological Activity of Metallo-BZD Derivatives

As mentioned above, BZDs are important drugs that are widely used in the treatment of a series of neurological pathologies. However, these drugs and derivatives have also demonstrated potential pharmacological activity and can act, for instance, against cancer [52], allergy [53], bacterial [54], fungal infections [55], and also as targeting agents for the cholecystokinin 2 receptor (CCK2R), which is overexpressed in some people [56,57]. Although reviews have been published on the different pharmacological activities of benzodiazepine-based compounds and their mechanism of action [4,5], studies in the field of drug discovery based on their metallo-derivatives are scarce.

The first publications describing pharmacological tests with metal complexes bearing BZD-like drugs as ligands appeared in the early 2000s, and were not related to neurological aspects. Visnjevac and coworkers [43] evaluated the in vitro cytostatic and antiviral activity of adducts of Cu(II), Zn(II) and Pd(II) and chiral 3-substituted 5-(2′-pyridyl)-1,4-benzodiazepin-2-one derivatives (see Table 1, Entries 12 and 13) and compared the results with those obtained for free ligands. Although the complexes and the free ligands did not present good activity, these studies appear to be the first aimed at evaluating the biological activity of metallo-BZDs.

Based on the anticancer activity previously demonstrated for BZDs, Spencer and coworkers, in 2009, carried out in vitro studies with palladacycles derived from 1,4-benzodiazepin-2-ones (see Table 1, Entry 18) using human cancer cells. They conducted assays to determine the inhibitory activity of cathepsin B bovine, an enzyme involved in cancer-related events [48,49]. The palladacycles with two metal centers linked by a dppe bridge and with an iso-Pr substituent on C(3) in the benzodiazepine ring showed promising results in terms of cytotoxicity and inhibition of the bovine cathepsin B, with IC50 values in the low mM range.

More recently, Barros and coworkers carried out, for the first time, anticonvulsant studies in vivo with palladacycle-derived BZD drugs (diazepam) [50]. The anticonvulsant potential of the dimeric complexes (see Table 1, Entry 19) was evaluated using two animal models (mice): pentylenetetrazole (PTZ)- and picrotoxin (PTX)-induced convulsions. The dimer with an acetate bridge significantly increased latency and protected the animals against convulsions induced by PTZ and PTX, while the analogous chloro derivative was effective (p b 0.01) only in the PTZ model. These effects appear to be mediated through the GABAergic system. A possible mechanism of action of the Pd(II)-diazepam-H complexes was also confirmed with the use of flumazenil (FLU), a GABAA-benzodiazepine receptor complex site antagonist. This was the first report of the anticonvulsant properties of metallo-BZD drugs, opening a new area in the development of novel drugs for the treatment of neurological pathologies.

## 4. Conclusions

Benzodiazepines represent an important family of compounds in modern medicinal chemistry, possessing a wide spectrum of biological activity with applications in several different fields. The enormous amount of data reported regarding the molecular structure–activity relationship of BZD drugs allows efficient approaches to the design of more effective drugs. This review provides details of studies that confirm the formation of organometallic complexes derived from benzodiazepines, with several metals coordinated to the organic portion. This characteristic allows alternative compounds and candidates for new drugs to be obtained. However, although the rich chemistry of BZD drug molecules allows the incorporation of metal atoms into their molecular skeleton (as discussed in this review paper), few examples have been synthesized and not all of them have been evaluated to determine their pharmacological potential. This review highlights the need for more research groups to explore the potential of the pharmacological activity of metallo-BZD compounds in the near future.

## Figures and Tables

**Table 1:** Examples of organometallic and coordination compounds containing BZDs as ligands, illustrating the chronological evolution of the theme.

Entry	Compound	Reference
1	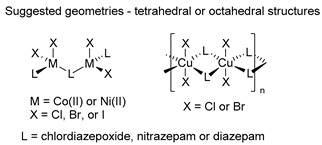	Preti and Tosi, 1976 [26] and 1979 [27].
2	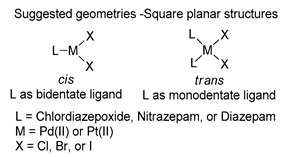	Preti and Tosi, 1976 [28].
3	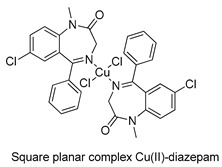	Mosset et al., 1980 [29].
4	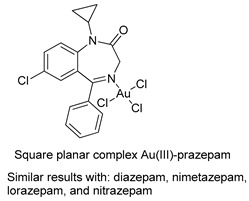	Minghetti et al., 1982 [30] and 1984 [31]
5	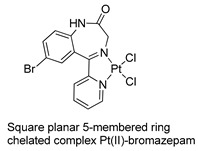	Benedetti et al., 1987 [32].
6	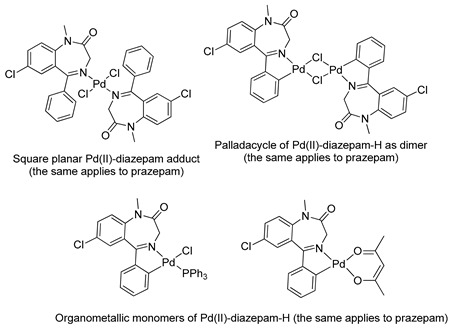	Cinellu et al., 1988 [35].^a^
7	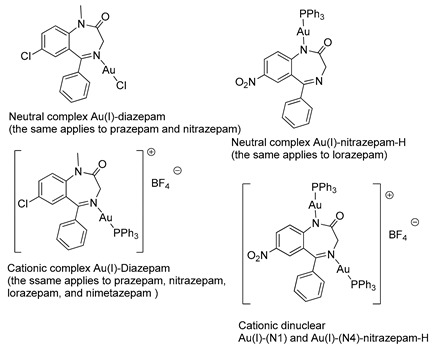	Cinellu et al., 1990 [37]
8	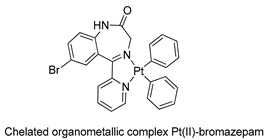	Aversa et al., 1991 [38].
9	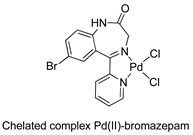	Antolini et al., 1992 [33]
10	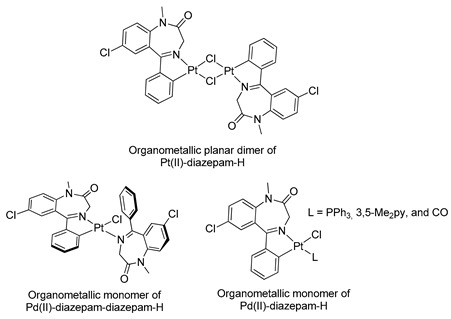	Stoccoro et al., 1994 [40].^a^
11	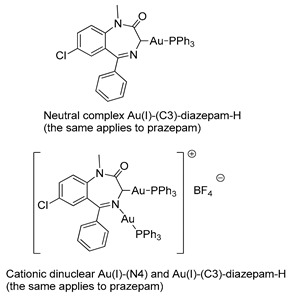	Minghetti et al., 1998 [41].
12	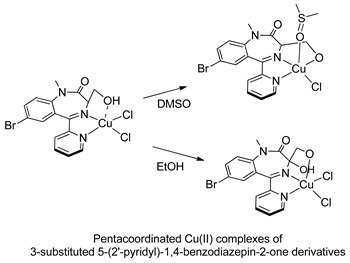	Visnjevac et al., 2001 [42].
13	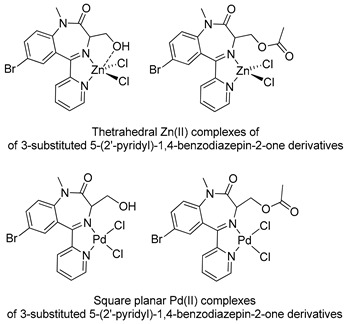	Visnjevac et al., 2002 [43].
14	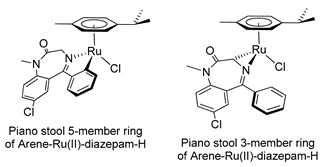	Pérez et al., 2002 [44].
15	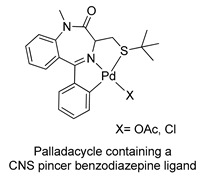	Spencer et al., 2005 [45].
16	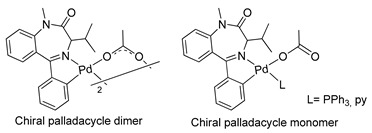	Spencer et al., 2008 [47].^a^
17	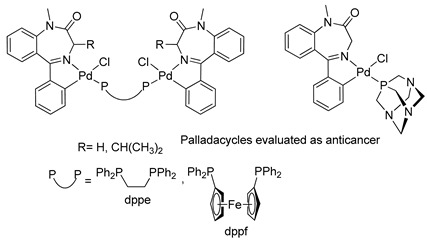	Spencer et al., 2009 [48,49].
18	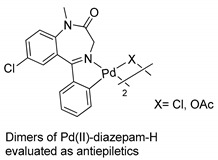	Barros et al., 2016 [50].^a^

^a^ The dimers are a mixture of their cisoid or transoid forms [50,51].
